# Oxygen-dependent partitioning of acetate assimilation via Acs and AckA-Pta pathways in *Pseudomonas aeruginosa*

**DOI:** 10.1128/jb.00182-26

**Published:** 2026-07-27

**Authors:** M. E. Watkins, K. V. Tonapi, C. M. VanDrisse

**Affiliations:** 1 Department of Genetics, University of Georgia, Athens, Georgia, USA; University of Virginia School of Medicine, Charlottesville, Virginia, USA

**Keywords:** metabolism, oxygen-dependent regulation, acetate kinase (AckA), acetyl-CoA synthetase (Acs), *Pseudomonas aeruginosa*, acetate metabolism

## Abstract

*Pseudomonas aeruginosa* is a metabolically versatile pathogen that thrives in host environments characterized by gradients of oxygen and nutrient availability. Although acetate is an abundant carbon source in chronic infections, the mechanisms governing *P. aeruginosa* acetate assimilation remains poorly understood. In many bacteria, acetate utilization occurs via the low-carbon-flux acetyl-CoA synthetase (Acs) or the high-carbon-flux acetate kinase/phosphotransacetylase (AckA-Pta) pathways. Here, we show that in *P. aeruginosa*, the above-mentioned pathways assimilate acetate as a function of oxygen availability, not as a function of carbon concentration. Our growth data demonstrate that AcsA is required for robust growth on acetate under oxic conditions, whereas the AckA-Pta pathway is essential for robust growth on acetate under anoxic conditions. Complementation experiments reveal that both pathways are functionally capable of acetate assimilation regardless of oxygen presence, indicating that pathway usage is primarily controlled by transcriptional regulation. Consistent with this, RT-qPCR analysis shows that *acsA* expression was elevated under oxic conditions, while *ackA* and *pta* were upregulated in the absence of oxygen. Despite these regulatory differences, kinetic analyses demonstrated that Acs and AckA from *P. aeruginosa* exhibit substrate affinities and catalytic efficiencies comparable to those of *Salmonella enterica*, indicating that the ability of both pathways to support *P. aeruginosa* growth across acetate concentrations is not due to altered enzyme kinetics like in other organisms. Together, these findings establish a new model in which oxygen availability, rather than acetate concentration, governs acetate assimilation in *P. aeruginosa*, with important implications for metabolic adaptation during infection.

**IMPORTANCE:**

Recent work has highlighted metabolic regulation as a driver of virulence, yet how *P. aeruginosa* metabolizes key metabolites found at infection sites remains uncharacterized. *P. aeruginosa* has a strong preference for acetate over other conventional carbon sources such as glucose, and acetate is found at millimolar concentrations in hosts. However, how *P. aeruginosa* regulates pathways involved in acetate assimilation remains largely unknown. Because steep oxygen gradients exist in host niches, where *P. aeruginosa* encounters acetate, understanding how acetate assimilation pathways are regulated via oxygen tension is essential for identifying spatial vulnerabilities at infection sites.

## INTRODUCTION

*Pseudomonas aeruginosa* is an opportunistic pathogen responsible for a wide range of infections in individuals with cystic fibrosis, burn wounds, or chronic wounds (1). Successful colonization of *P. aeruginosa* within these environments relies heavily on metabolic adaptability as bacteria encounter dynamic gradients of nutrient and oxygen availability (2). As chronic infections develop, oxygen is depleted through microbial respiration and host inflammatory responses, causing a shift in bacterial metabolic strategies (3, 4). At the same time, the identities and availability of carbon sources change as polymicrobial communities degrade host-derived substrates (5). Together, these factors generate a complex metabolic landscape to which pathogens must adapt to persist during infection. Understanding the interplay of metabolic pathways used by *P. aeruginosa* and how they are regulated is essential for understanding its success as a pathogen.

**Fig 1:**
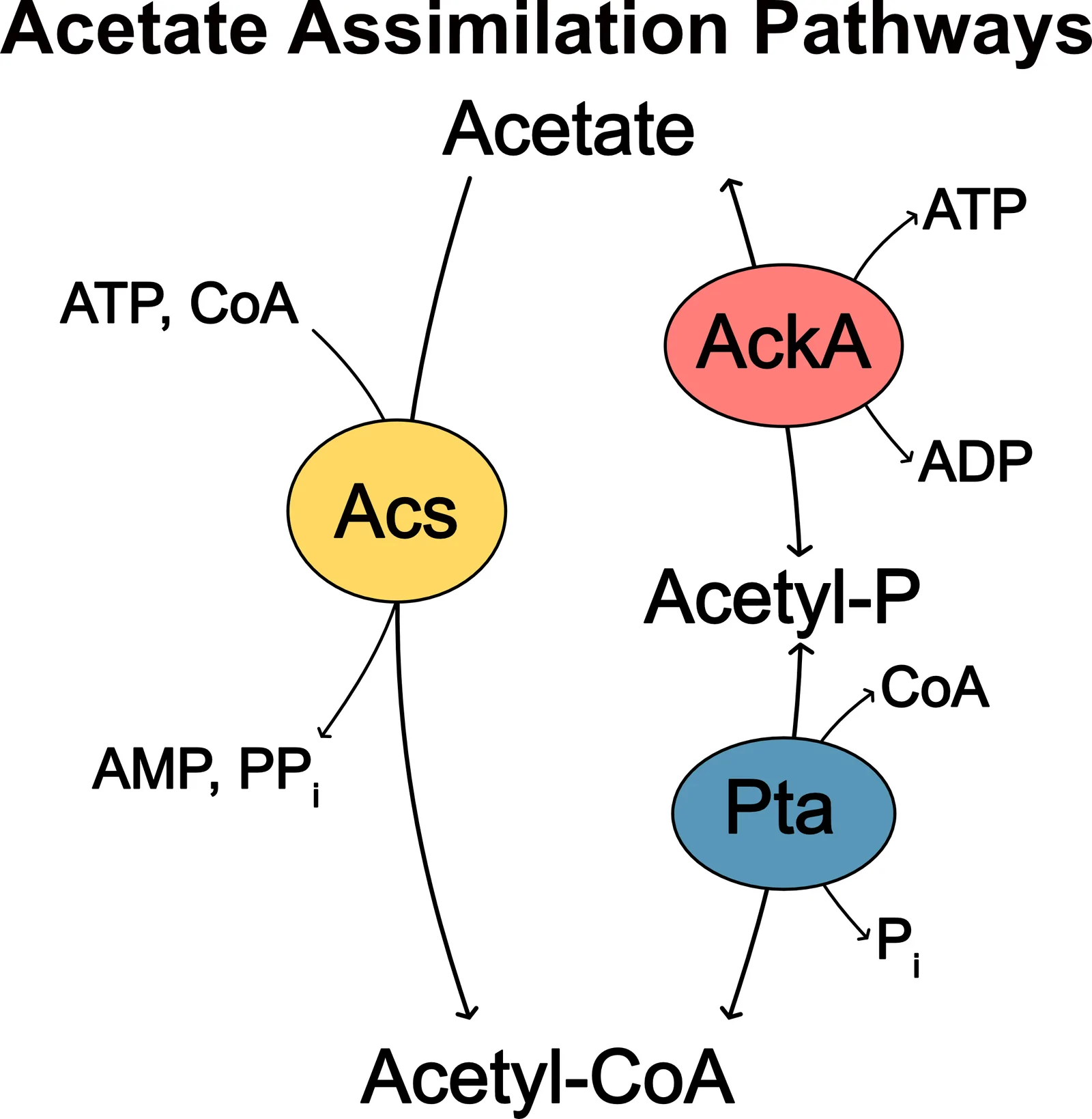
Acetate metabolism in enteric bacteria. Left: Acs activates acetate to acetyl-CoA using ATP and CoA at low concentrations of acetate. Right: AckA converts acetate to acetyl-phosphate using ATP, while Pta converts acetyl-P to acetyl-CoA at high concentrations of acetate. The reaction is reversible.

With this in mind, even though *P. aeruginosa* can metabolize diverse carbon sources, it displays an unusually strong preference for acetate (6, 7), which is present at millimolar levels in chronic infection environments due to mucin-degrading polymicrobial communities and immune cells that alter their metabolism during infections (5, 8–12). Despite this fact, how *P. aeruginosa* regulates varying acetate assimilation pathways is largely unknown. In enteric model organisms, such as *S. enterica*, acetate is catabolized to acetyl-CoA through two distinct pathways. At low acetate concentrations, the AMP-forming acetyl-CoA synthetase (Acs) pathway is primarily used, driven by the enzyme’s high affinity for acetate and increased transcription of *acs* under nutrient limitation (13) (Fig. 1, left). At high acetate levels, flux shifts to the lower-affinity acetate kinase and phosphotransacetylase (AckA-Pta) pathway, which generates acetyl-CoA via an acetyl-phosphate intermediate (13) (Fig. 1, right). In *P. aeruginosa*, it is unknown whether this same delineation exists, although others have shown that the deletion of *acs* is inhibitory to aerobic growth on acetate (20 mM) (5).

The *P. aeruginosa* genome encodes two Acs homologs, AcsA (PA14_52800) and AcsB (PA14_62630), both predicted to convert acetate into acetyl-CoA through an AMP-forming reaction. Prior studies have shown that deletion of *acsA* impairs aerobic growth on 20 mM acetate (5), but the role of *acsB* has not been explored. Homologs of AckA (PA14_53470) and Pta (PA14_53480) are also present in *P. aeruginosa*, but existing analyses have focused solely on the role of this pathway in a form of anaerobic survival known as facilitated fermentation (14, 15). Under this mode of metabolism, acetate and ATP are generated from acetyl-CoA as a form of energy conservation, ensuring survival in anoxic environments, where terminal electron acceptors are limited (16). As such, it has been shown via microarrays that *ackA-pta* genes are transcribed abundantly during anaerobic growth (14). No studies have examined whether *P. aeruginosa* uses AckA-Pta for forward acetate assimilation or how the Acs and AckA/Pta pathways are regulated.

This study seeks to examine the regulation of acetate assimilation in *P. aeruginosa* using growth analyses, enzymology, and transcriptional analyses. We highlight that the two acetate assimilation pathways in *P. aeruginosa* are delineated by the presence of oxygen: the AcsA pathway supports growth on acetate under oxic conditions, whereas the AckA-Pta pathway functions in the absence of oxygen. These growth phenotypes correlate with transcriptional changes measured by RT-qPCR, and enzymatic analyses reveal distinct apparent affinities for acetate for AcsA and AckA. These data highlight the first observation of the AckA-Pta pathway in *P. aeruginosa* functionally proceeding in a catabolic direction. Additionally, our data show for the first time in a bacterial species that the functionality of the AcsA and AckA-Pta pathways is directly linked to the concentration of oxygen in the environment, rather than acetate concentration. This metabolic partitioning has implications for understanding *P. aeruginosa* metabolism in host environments, where oxygen gradients and acetate concentrations vary across infection sites and biofilm structures.

## RESULTS

### AcsA and AckA-Pta support acetate assimilation under distinct oxygen conditions

Based on prior characterizations of acetate metabolism in *Escherichia coli* and *S. enterica*, we chose to assay acetate assimilation phenotypes using two distinct concentrations. Others have shown in *E. coli* that acetate is secreted as a form of carbon overflow metabolism, and once acetate accumulates in the extracellular environment to a concentration of 5–10 mM or higher, metabolism shifts from acetate production to consumption (17). With this in mind, genetic studies have shown that ∆*acs* mutants from enteric organisms exhibit growth defects at low acetate concentrations (e.g., ~10 mM), where high-affinity assimilation is required, whereas ∆*ackA-pta* mutants are impaired at higher acetate concentrations (≥30 mM) (17). In our experiments, we chose 10 mM and 60 mM acetate to represent conditions at and well above this transition point. Concentrations lower than 10 mM were not assayed due to low growth yields, and concentrations in excess of 60 mM were not tested due to acid stress associated with high acetate concentrations, as protonated acetate can diffuse across the membrane and dissociate intracellularly, leading to cytoplasmic acidification and disruption of the proton motive force (18).

**Fig 2:**
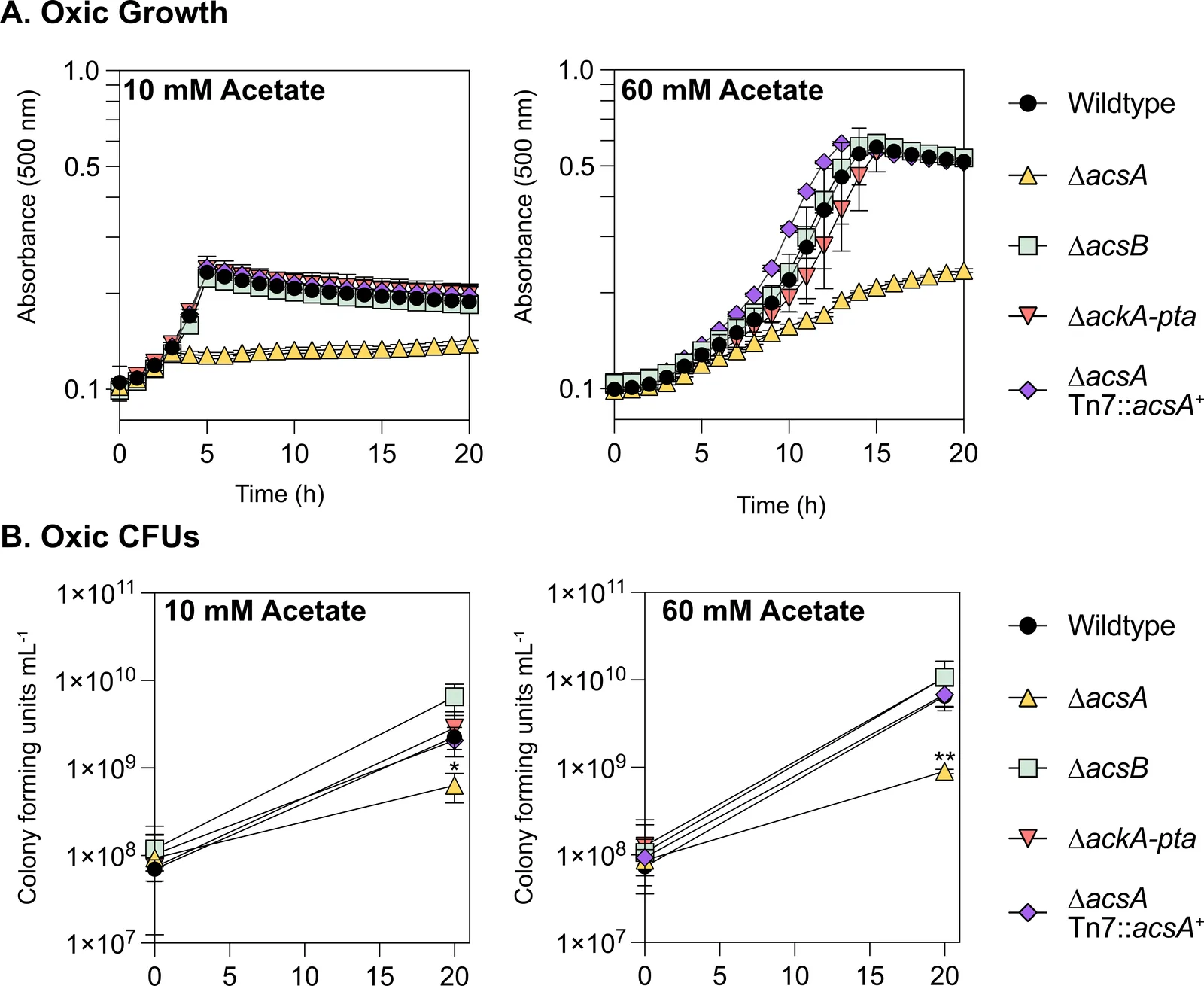
Oxic growth of *P. aeruginosa* strains on low and high acetate concentrations. (**A**) *P. aeruginosa* was subcultured into MOPS minimal medium with rhamnose (1 mM), nitrate (20 mM), and either 10 mM acetate (left) or 60 mM acetate (right), and absorbance was monitored over time in an EPOCH2 Biotek plate reader (see Materials and Methods for details). (**B**) At time zero and end points, cells were serially diluted and plated for determining colony-forming units per mL. * = *P*-value < 0.05 and ** = *P*-value < 0.01. Strains (legends on right) were grown in triplicate with error bars in panels A and B representing 95% confidence intervals.

To determine the catabolic roles of the AcsA, AcsB, and AckA-Pta pathways, we generated in-frame deletions of *acsA*, *acsB*, and the *ackA-pta* operon. We then assessed the growth capabilities of these *P. aeruginosa* strains on low (10 mM) and high (60 mM) concentrations of acetate. Based on precedent of Acs and AckA-Pta functions in other model organisms described above (17), we hypothesized that an *acs* deletion would not grow on low acetate and an *ackA-pta* deletion would not grow on high acetate (19, 20). Unexpectedly, ∆*acsA* had a growth defect on both concentrations of acetate, while ∆*ackA-pta* and ∆*acsB* grew on all concentrations of acetate (Fig. 2A), which was also mirrored in cell counts (Fig. 2B). The phenotype of ∆*acsA* was complemented successfully through the introduction of *acsA* downstream of a rhamnose-inducible promoter at the Tn7 site of *P. aeruginosa* (Fig. 2). The results with *P. aeruginosa* ∆*acsA* contrasted with the canonical model derived from enteric bacteria, in which Acs primarily supports growth at low acetate concentrations, and suggested that AcsA plays a broader role in acetate metabolism in *P. aeruginosa* under these conditions.

**Fig 3:**
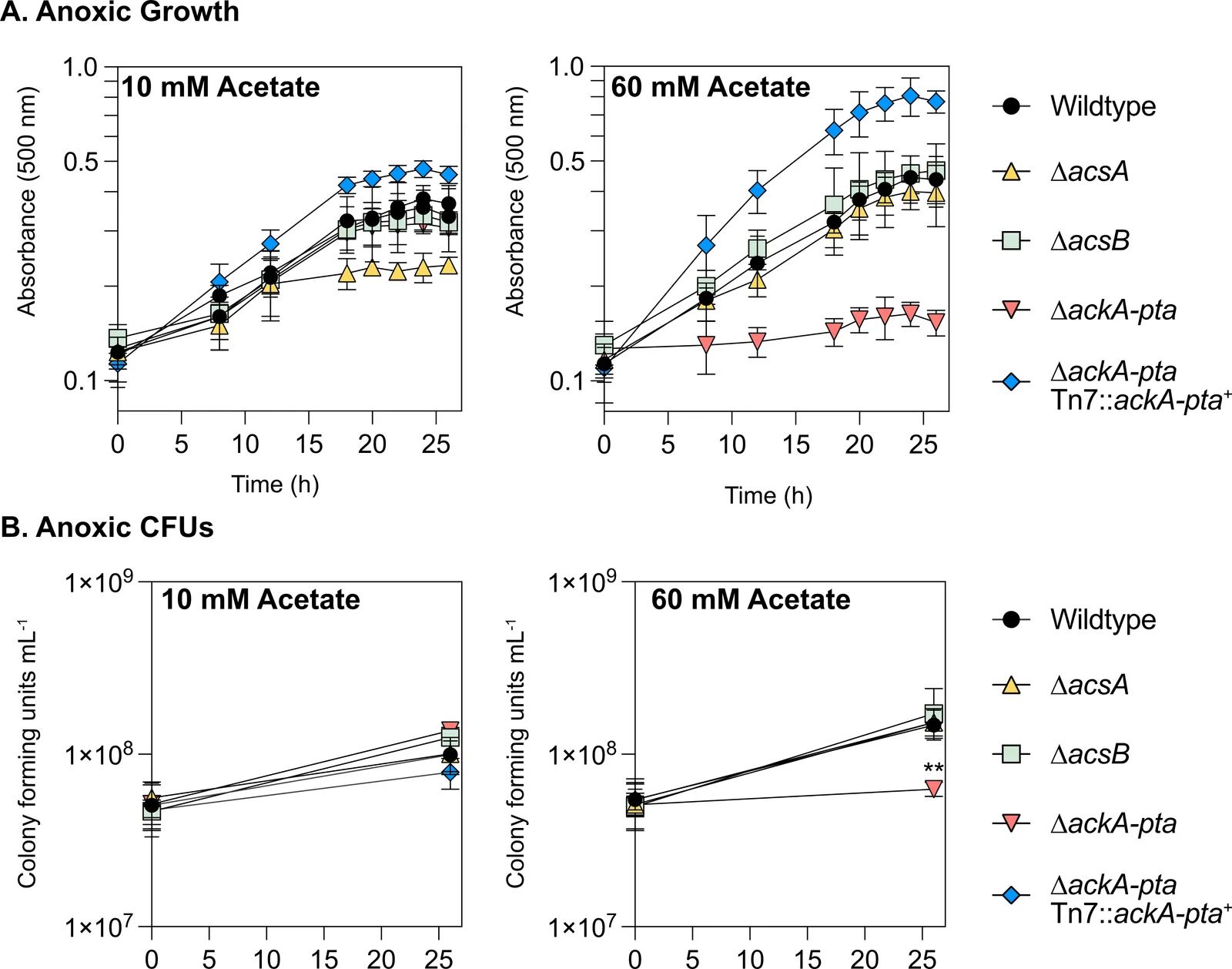
Anoxic growth of *P. aeruginosa* strains on low and high acetate concentrations. (**A**) *P. aeruginosa* was subcultured into MOPS minimal medium with rhamnose (1 mM), nitrate (20 mM), and either 10 mM acetate (left) or 60 mM acetate (right). Culture tubes were vacuumed and degassed, and absorbance was monitored over time using a Spectronic 20D+ spectrophotometer. (**B**) At time zero and end points, cells were serially diluted and plated for determining colony-forming units per mL. ** = *P*-value < 0.01. Strains (legends on right) were grown in triplicate with error bars in panels A and B representing 95% confidence intervals .

Others have found that the transcription of *ackA-pta* in *P. aeruginosa* is upregulated in the absence of oxygen (14), so we attempted to grow ∆*acsA*, *∆acsB*, and ∆*ackA-pta* anoxically on 10 mM and 60 mM acetate. At 10 mM acetate under anaerobic conditions, we saw a slight growth defect only with ∆*acsA*; however, this defect was not reflected in the cell counts (Fig. 3A). We therefore did not detect clear strain-specific phenotypes under anaerobic conditions at this concentration, potentially due to sensitivity limitation of tube-based measurements combined with low carbon availability. However, at 60 mM acetate, ∆*acsA* and ∆*acsB* showed no difference from wild type, while the ∆*ackA-pta* strain had a growth defect (Fig. 3A), which was also observed via cell counts (Fig. 3B). Expression of *ackA-pta* from a rhamnose-inducible promoter at the Tn7 site restored the anoxic ∆*ackA-pta* phenotype on acetate (Fig. 3A). While the ∆*ackA-pta* Tn7::*ackA-pta^+^*strain appeared to reach a higher OD than wild type, this was not reflected in the cell counts (Fig. 3B). The phenotype with ∆*ackA-pta* differs from that observed under oxic conditions, where ∆*ackA-pta* did not have a measurable growth defect, indicating that the requirement for AckA-Pta was dependent on oxygen availability rather than acetate concentration alone. The absence of a phenotype for the ∆*acsB* strain under any condition tested suggested that AcsB did not contribute substantially to acetate metabolism in these conditions. This finding raises the possibility that AcsB serves an alternative function or is differentially regulated in *P. aeruginosa*. Together, these results showed that acetate assimilation in *P. aeruginosa* is controlled by oxygen availability under the conditions tested, with AcsA supporting growth under oxic conditions and AckA-Pta functioning under anoxic conditions.

### AckA-Pta and AcsA are functionally capable of acetate activation

**Fig 4:**
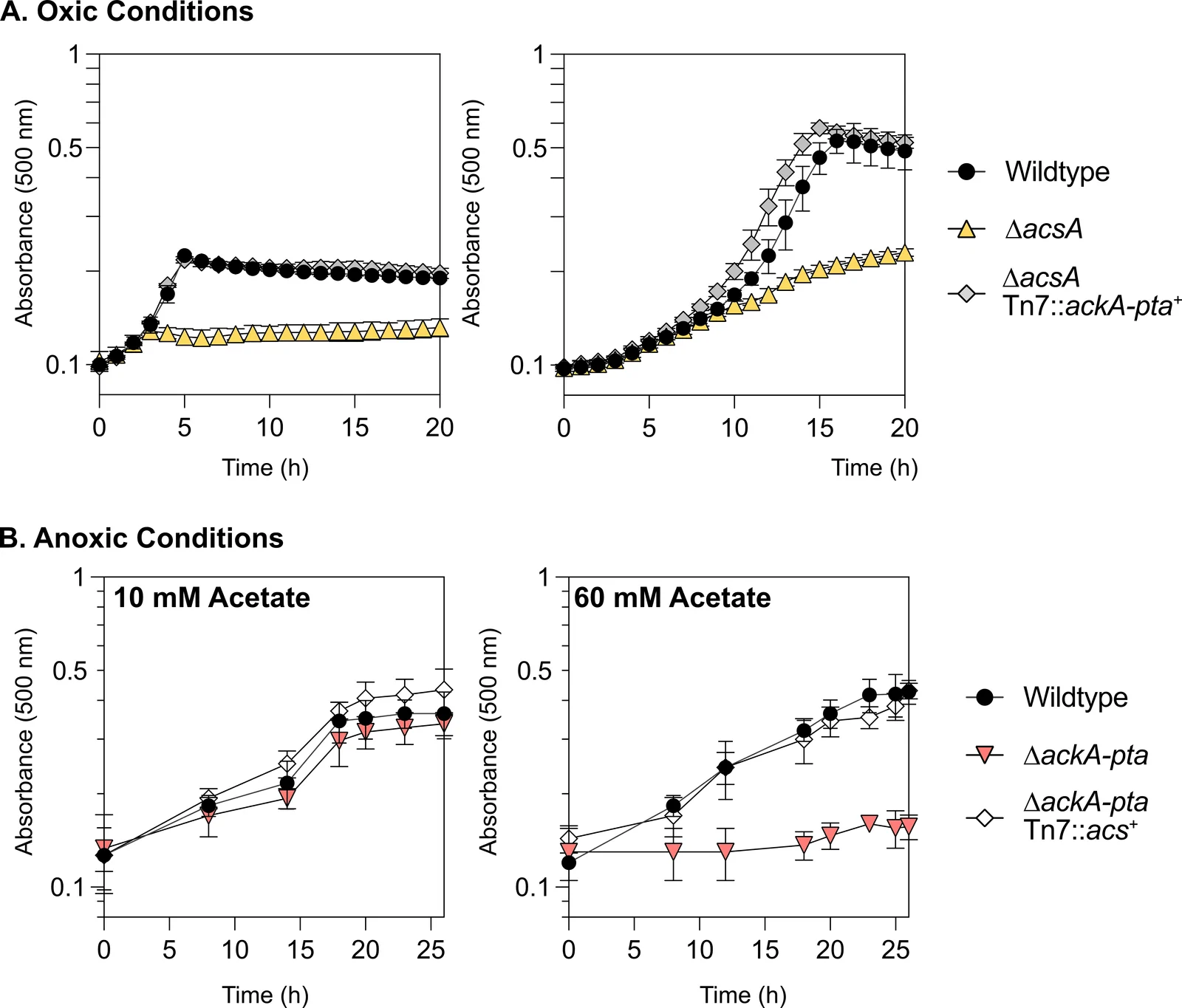
Complementation of ∆*acs* and ∆*ackA-pta* phenotypes with *ackA-pta*+ or *acs*+. (**A**) *P. aeruginosa* was subcultured into MOPS minimal medium with rhamnose (1 mM), nitrate (20 mM), and either 10 mM acetate (left) or 60 mM acetate (right), and absorbance was monitored over time using an EPOCH2 Biotek plate reader (see Materials and Methods for details). (**B**) *P. aeruginosa* was subcultured into MOPS minimal medium with rhamnose (1 mM), nitrate (20 mM), and either 10 mM acetate (left) or 60 mM acetate (right). Culture tubes were vacuumed and degassed, and absorbance was monitored over time using a Spectronic 20D+ spectrophotometer. A and B. Strains (legends on right) were grown in triplicate, with error bars representing (**A and B**) Strains (legends on right) were grown in triplicate, with error bars representing 95% confidence intervals.

The observed growth phenotypes on acetate were strongly dependent on the presence or absence of oxygen. We therefore asked whether this oxygen dependence reflected intrinsic differences in enzyme activity under aerobic versus anaerobic conditions, or instead differences in the presence of the corresponding gene products. To distinguish between these possibilities, we expressed each pathway from an inducible locus at the Tn7 site in the corresponding deletion background under both conditions. Induction of *ackA-pta* from a neutral site in the chromosome in a ∆*acsA* background restored robust growth on 10 mM and 60 mM acetate under oxic conditions (Fig. 4A), indicating that *ackA-pta* genes were likely not expressed under the conditions tested, but their protein products could still activate acetate to acetyl-CoA. Therefore, the growth defect of the ∆*acsA* mutant was not due to lack of AckA-Pta function in the presence of oxygen.

Conversely, induction of *acsA* in the ∆*ackA-pta* background led to robust growth anoxically on 60 mM acetate (Fig. 4B). Similarly, this finding indicated that AcsA could support acetate metabolism under anoxic conditions when expressed, despite not contributing in the wild-type strain. Together, these results demonstrated that both pathways initiate acetate metabolism, and that their differential usage is governed by an as-yet undefined regulatory mechanism. These findings suggested that the oxygen-dependent phenotypes observed in the deletion strains arose from condition-specific regulation of pathway expression rather than intrinsic differences in enzyme activity or stability.

### Oxygen regulates transcription of acetate assimilation pathways

Previous work using transcriptomics via microarrays of *ackA-pta*, together with biochemical assays of Pta activity under anaerobic growth, demonstrated that this pathway is upregulated in the absence of oxygen (14). Through these studies, it has been shown that *ackA-pta* expression increases in an Anr-dependent manner (14), a transcriptional activator with an iron-sulfur center that functions in the absence of oxygen. Given our observation that AcsA supports growth under oxic conditions while AckA-Pta is required under anoxic conditions, we reasoned that these phenotypes may be driven by differential transcription of the corresponding pathways. Specifically, we hypothesized that *acsA* expression would be elevated under oxic conditions, whereas *ackA-pta* expression would be preferentially upregulated in the absence of oxygen.

**Fig 5:**
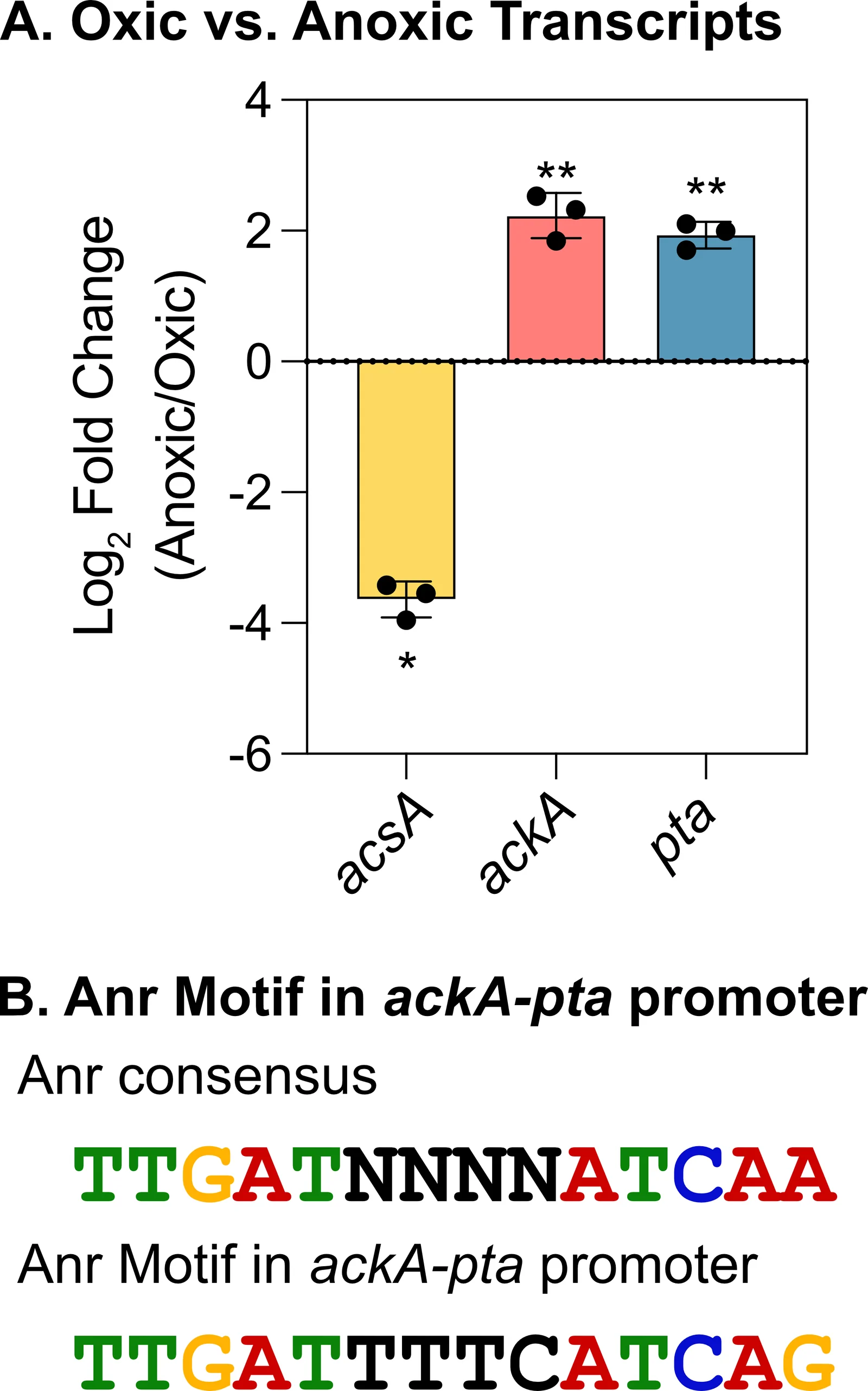
RT-qPCR of acetate assimilation genes grown oxically or anoxically. (**A**) Transcript levels of *acs*, *ackA*, and *pta* under anoxic conditions are plotted as log_2_ fold change relative to oxic conditions. Points indicate biological replicates, and bars represent mean ± SD. Statistical analyses were performed on normalized relative expression values using unpaired two-tailed Student's *t*-tests comparing oxic and anoxic conditions for each gene. Transcripts were normalized to the *rpoS* gene of PA14. * = *P* < 0.05 and ** = *P* < 0.01. (**B**) Alignment of the conserved Anr consensus binding motif (TTGAT-N4-ATCAA) with a predicted Anr-binding site identified 196 bp upstream of the *ackA-pta* promoter using FIMO analysis (*P* = 3.59 × 10-5, *q* = 0.03)

To test this hypothesis, we purified RNA from wild-type *P. aeruginosa* grown on 60 mM acetate under oxic or anoxic conditions and quantified transcript levels by RT-qPCR. Our log_2_ fold-change analysis demonstrated that *acsA* transcript abundance decreased by approximately 3.7 log_2_ folds, whereas *ackA* and *pta* transcript abundance increased by approximately two log_2_ folds under anoxic conditions relative to oxic conditions (Fig. 5A). These transcriptional patterns were consistent with our observed growth phenotypes, in which Acs supported acetate assimilation under oxic conditions and AckA-Pta functions under anoxic conditions. These results indicated that oxygen availability regulates transcription of the two acetate assimilation pathways and determines which pathway supports growth on acetate. As stated above, the regulation of the *ackA-pta* promoter has been shown to be Anr-dependent (14). Consistent with this observation, a FIMO (21) analysis identified a conserved putative Anr-binding motif (22) 196 bp upstream of the *ackA-pta* operon promoter (*P =* 3.59 × 10^−5^, *q* = 0.03) (Fig. 5B). We performed a similar FIMO analysis of the *acs* promoter using known *P. aeruginosa* transcription factor binding motifs (23); however, no statistically significant motif matches were identified. This observation may indicate regulation by an as-yet unidentified transcription factor.

### Acs and AckA display conserved acetate affinities in *P. aeruginosa* and *Salmonella*

Because acetate assimilation pathways in enteric bacteria are thought to be partitioned by enzyme affinity (17), we sought to measure the kinetic parameters of Acs and AckA for acetate from *P. aeruginosa* and *S. enterica*. This experiment was motivated by our observation that *P. aeruginosa acsA* and *ackA-pta* mutants displayed oxygen-dependent growth yet grew in medium supplemented with low and high acetate concentrations. We therefore wondered whether differences in enzyme affinity could explain the distinct acetate growth phenotypes observed in *P. aeruginosa*. To investigate this, we measured the kinetic parameters of Acs and AckA from *P. aeruginosa* and *S. enterica* via an NADH-coupled spectrophotometric assay to compare enzyme affinities. Purified enzymes were assayed across a range of acetate concentrations to determine *K_m_*, *k*_cat_, and catalytic efficiency (*k*_cat_/*K_m_*). We analyzed technical quadruplicates for each concentration of acetate, completed for three independent experiments, with averages and standard deviation reported.

**Fig 6:**
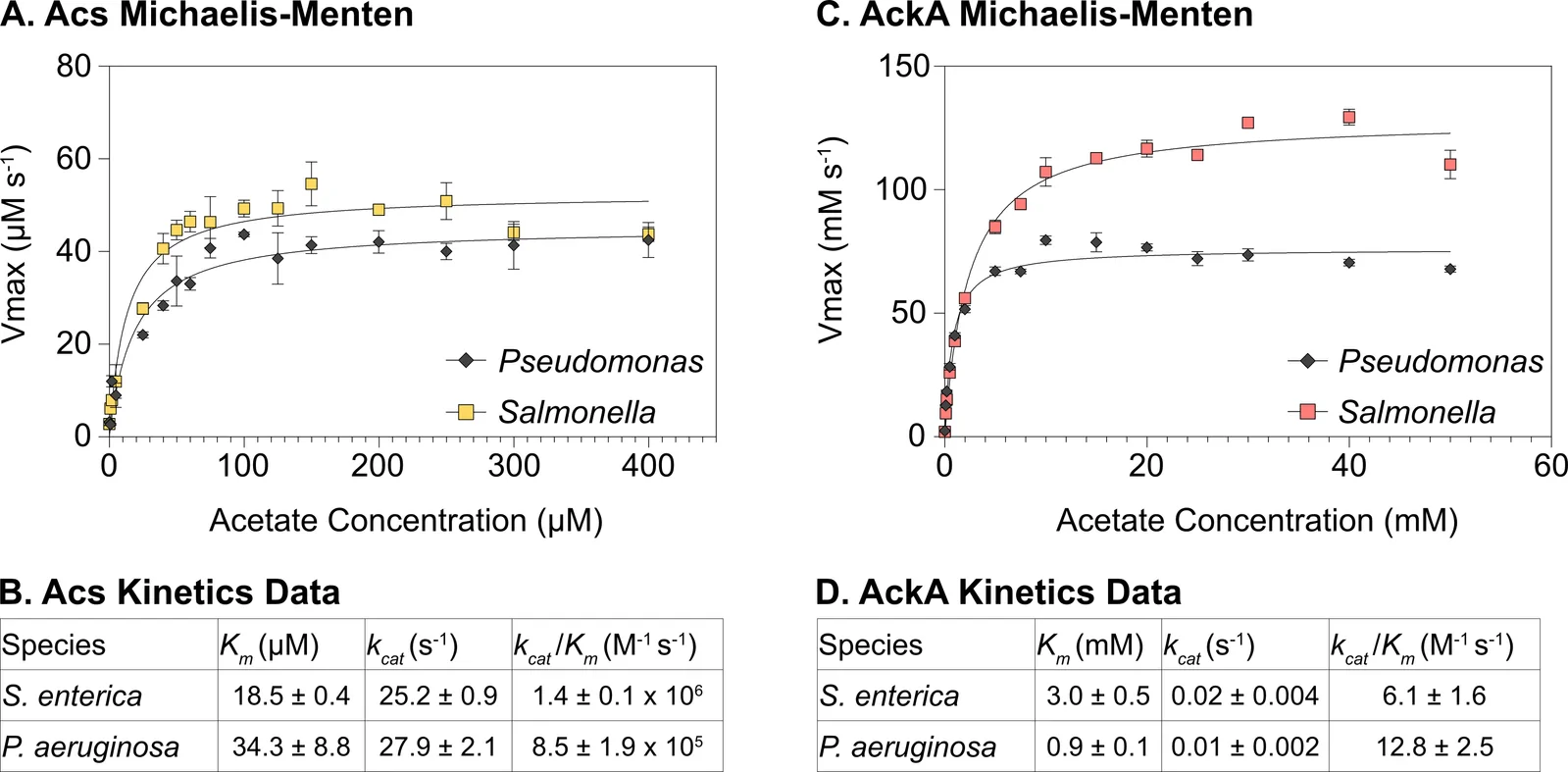
Michaelis–Menten data for Acs and AckA. (**A**) Michaelis–Menten kinetics of *P. aeruginosa* and *S. enterica* Acs were calculated by plotting *V*_max_ values against acetate concentration, as indicated on the x-axis. Error bars represent the standard deviation of the mean of triplicates. (**B**) Average values with standard deviation for *K*_*m*_, *k*_cat_, and *k*_cat_/*K*_*m*_ from three independent experiments (graph in panel A is representative graph of one experiment). (**C**) Michaelis–Menten kinetics of *P. aeruginosa* and *S. enterica* AckA (3 µM) were calculated by plotting *V*_max_ values against acetate concentration, as indicated on the x-axis. Error bars represent standard deviation of the mean of triplicates. (**D**) Average values with standard deviation for *K*_*m*_, *k*_cat_, and *k*_cat_/*K*_*m*_ from three independent experiments (graph in panel A is representative graph of one experiment).

Under the conditions tested, AcsA from *P. aeruginosa* exhibited a *K_m_* for acetate of 34.3 µM, a *k*_cat_ of 27.2 s^−1^, and a catalytic efficiency of 8.5 × 10^5^ M^−1^s^−1^. In comparison, Acs from *S. enterica* displayed a *K_m_* of 18.5 µM, a *k*_cat_ of 25.2 s⁻¹, and a catalytic efficiency of 1.4 × 10^6^ M^−1^s^−1^ (Fig. 6A and B). Although the *K_m_* of *P. aeruginosa* AcsA was modestly higher than that of the *S. enterica* enzyme, both enzymes exhibited micromolar affinity for acetate. Notably, these Acs enzymes appeared to have a higher affinity for acetate than Acs from *E. coli*, which has a *K_m_* value near 200 µM (24). AckA enzymes from both *P. aeruginosa* and *S. enterica* exhibited lower affinities for acetate compared to their respective Acs (Fig. 6C and D). These values were consistent with the established kinetics of AckA, with *K_m_* values in the millimolar range (7–10 mM) (17). Together, these results indicated that Acs and AckA maintain conserved acetate affinity rules across species, with Acs functioning as a high-affinity enzyme and AckA operating in the lower-affinity millimolar range.

## DISCUSSION

In enteric bacteria, acetate metabolism is classically described by the “acetate switch,” in which cells transition from acetate excretion during rapid growth on preferred carbon sources to acetate assimilation as those substrates are depleted (17). In this paradigm, the two acetate assimilation pathways are thought to be partitioned primarily by substrate availability, with the high-affinity Acs pathway functioning at low acetate concentrations and the lower-affinity AckA-Pta pathway operating when acetate is abundant (17). While genetic analyses have demonstrated that these pathways are collectively required for growth on acetate, studies directly testing whether the pathways are functionally interchangeable when expressed under the same conditions are lacking. In contrast to enteric bacteria, our results indicate that in *P. aeruginosa*, pathway usage is determined by oxygen availability rather than acetate concentration. Transcriptional regulation partitions pathway usage such that Acs functions under oxic conditions, whereas AckA-Pta is utilized in the absence of oxygen. Others have shown that deletion of the *P. aeruginosa* anaerobic activator Anr decreases *ackA-pta* transcripts (14), but whether this effect is direct or indirect is unknown. However, a scan of the 500 base pairs upstream of the *ackA* start codon displayed a binding motif for Anr (25) at −196, but assays of direct Anr binding to this promoter will require further analysis. The *acs* gene in enteric bacteria is regulated by the catabolite repression protein (Crp) in response to glucose availability (26). In contrast, *P. aeruginosa* does not employ Crp-mediated catabolite repression. To date, transcriptional regulators controlling *acsA* expression in *P. aeruginosa* have not been identified.

Despite the clear oxygen-dependent delineation of AcsA and AckA-Pta pathway usage, biochemically, *P. aeruginosa* AcsA and AckA were kinetically similar to the homologous enzymes from *S. enterica.* Acs and AckA share approximately 66% and 46% sequence identity between these species, respectively. As such, Acs and AckA from *P. aeruginosa* and *S. enterica* exhibited similar orders of magnitude for *K_m_*, *k*_cat_, and catalytic efficiency. However, our data demonstrate that this intrinsic enzymatic activity is secondary to transcriptional regulation in response to oxygen availability. Thus, pathway usage is not dictated solely by substrate concentration but instead reflects a hierarchical regulatory system in which environmental cues override enzyme kinetics. These findings raise important questions regarding the conservation of acetate assimilation strategies across bacterial species. Because acetate metabolism has been primarily characterized in enteric organisms that thrive under majority anoxic environments, it remains unclear whether oxygen-dependent partitioning of AcsA and AckA-Pta represents a broader strategy among bacteria that encounter acetate in diverse ecological niches. Notably, despite substantial sequence similarity between the *P. aeruginosa* and *S. enterica* Acs/AckA enzymes, their regulations differ, highlighting the limitations of inferring metabolic mechanisms from sequence homology or annotation alone. This study therefore highlights the importance of experimental validation, such as through the use of genetic perturbation and growth analyses, in defining pathways rather than relying only on bioinformatic predictions.

**Fig 7:**
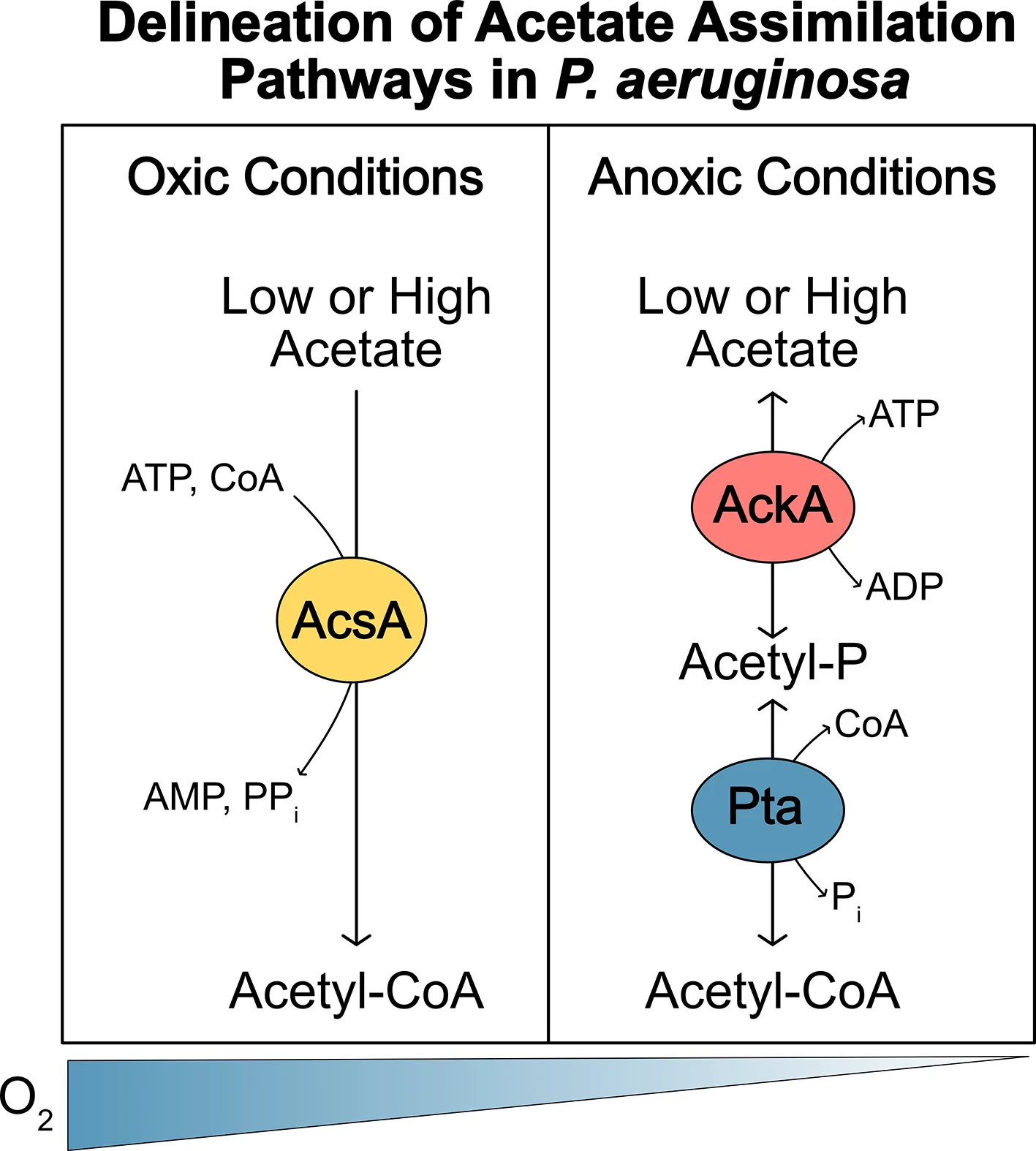
Proposed model for AcsA and AckA-Pta pathway delineation in *P. aeruginosa*. When oxygen levels are higher and acetate is available, the AcsA pathway is upregulated and favored. When anoxia is generated in the presence of acetate, the reversible AckA-Pta pathway is utilized.

These findings establish a fundamentally new model for regulation of acetate metabolism in bacteria. Because chronic infection sites generate oxygen gradients (27), it is possible that the unusual regulation of *P. aeruginosa* acetate assimilation may reflect adaptation to unique requirements of the host infection niche. Acetate is present in host tissues at low micromolar concentrations from endogenous mammalian metabolism (28), and sterile infections are typically hypoxic (29). We therefore hypothesize that during early colonization, *P. aeruginosa* utilizes the high affinity, oxygen-dependent Acs pathway for acetate assimilation (Fig. 7). Conversely, as time progresses, chronic infections have higher concentrations of acetate from polymicrobial activity (5), while oxygen consumption by both host and microbial cells generates zones of anoxia (3, 4). We speculate that under these conditions, *P. aeruginosa* would utilize the AckA-Pta acetate assimilation pathway (Fig. 7). A similar framework may apply within biofilms, where steep gradients of oxygen and metabolites are established from the surface to the interior (2). In addition to its metabolic role, the AckA-Pta pathway generates acetyl-phosphate, a high-energy intermediate that can act as a global phosphoryl donor to two-component regulatory systems (30). Thus, oxygen-dependent activation of this pathway may have broader regulatory consequences beyond acetate assimilation, potentially influencing virulence, stress responses, and biofilm development.

One limitation of this study is that pathway usage was inferred from growth phenotypes and transcriptional measurements under defined laboratory conditions, which may not fully capture the complexity of host environments. Oxygen gradients and acetate concentrations *in vivo* are dynamic and spatially heterogeneous, and future studies using infection models or defined gradients will be required to directly test how these pathways are utilized during infection. Additionally, while transcriptional differences strongly correlate with pathway usage, direct measurements of metabolic flux will be necessary to confirm these conclusions. Collectively, these observations highlight the fact that bacteria exhibit metabolic flexibility over the course of an infection, which should be considered when determining novel methods to undermine bacterial robustness *in vivo.* However, targeting acetate metabolism in *P. aeruginosa* as a therapeutic avenue may require consideration of oxygen availability, as inhibition of a single pathway may be insufficient across the diverse metabolic states encountered during infection.

## MATERIALS AND METHODS

### Bacterial strains and growth conditions

*Pseudomonas aeruginosa* UCBPP-PA14 and *Salmonella enterica* subsp. *enterica* serovar Typhimurium LT2 were used for analysis. *Escherichia coli* S-17 was used for plasmid construction, and *Escherichia coli* C41 *pat::kan^+^* (λDE3) was used for protein overexpression. Cells were grown at 37°C in lysogeny broth (LB, Difco) for propagation and in MOPS minimal medium supplemented with acetate (10 mM or 60 mM) (31) and potassium nitrate (20 mM), and L-Rhamnose (1 mM) as an inducer when required. Oxic cultures were grown with nitrate to ensure that phenotypes between oxic and anoxic conditions were due to oxygen presence rather than nitrate. When appropriate, antibiotics were used at the following concentrations: gentamicin (20 µg mL^−1^ for *E. coli* and 75 µg mL^−1^ for *P. aeruginosa*) and ampicillin (100 µg mL^−1^). All chemicals were purchased from Sigma Aldrich, with the exception of ampicillin (GoldBio).

### Strain construction and growth analysis

All strains in this study are listed in Table 1. In-frame deletions of *P. aeruginosa acs* and *ackA-pta* genes were constructed using allelic exchange (gentamicin selection followed by sucrose counterselection) as described elsewhere (32). Plasmids containing rhamnose-inducible genes were transformed and integrated into strains for complementation studies as described elsewhere (33). For oxic growth analyses, starter cultures were grown overnight at 37°C shaking in LB containing the appropriate antibiotic. Cultures (1% [vol/vol]) were used to inoculate 198 μL of fresh minimal medium placed in each well of a 96-well microtiter plate. Microtiter plates were incubated at 37°C inside the temperature-controlled chamber of an EPOCH2 microtiter plate reader (BioTek Instruments) with a 1°C gradient to prevent evaporation, and plates were continuously shaken using the linear shaking setting of the instrument. Time points were taken for 24 h every 30 min with data shown for every 1 h. Cell density was monitored at 500 nm, and data were analyzed using Prism v10 (GraphPad). For anoxic growth curves, minimal medium (5 mL) was inoculated with starting culture (starting OD of 0.1) in 18 mm Balch tubes. Tubes were stoppered and crimped, followed by three rounds of vacuuming and gas exchange (gas mix 85% N_2_, 10% CO_2_, 5% H_2_) using a manifold. Cultures were incubated in a 37°C shaker, and OD_500_ measurements were taken using a Spectronic 20D+ spectrophotometer. Oxic and anoxic growth studies were performed in triplicate in three independent experiments, with a representative growth curve shown with the 95% confidence interval of technical triplicates. Colony-forming units were calculated for time zero and endpoints by removing 10 µL of culture, followed by 10-fold dilutions that were plated using the drip plate method. In the case of oxic growth curves, culture/medium volume was added to the 96-well plate with 10 µL excess for CFU experiments.

**TABLE 1:** Bacterial strains used in this study

Strain	Relative genotype	Source^*a*^
*E. coli strains*		
*E. coli* S-17	RP4-2(Km::Tn7, Tc::Mu-1) *pro82*λ*pir recA1 endA1 thiE1 hsdR17 creC510*	(34)
*E. coli* C41 (λDE3)	*pka12::kan^+^ ompT* hsdS (r_B_m_B_) *gal* (lDE3)	Laboratory collection
*P. aeruginosa strains*		
CVD27	Wild-type *Pseudomonas aeruginosa* UCBPP-PA14	Laboratory collection
Derivatives of CVD27		
CVD586	∆*acsA* (PA14_52800)	
CVD588	∆*acsB* (PA14_62630)	
CVD172	∆*ackA-pta* (PA14_53470-80)	
CVD852	∆*acsA* Tn7::*acsA*	
CVD855	∆*acsA* Tn7::*ackA-pta*	
CVD853	∆*ackA-pta* Tn7::*acsA*	
CVD856	∆*ackA-pta* Tn7::*ackA-pta*	

^*a*^ Unless otherwise noted, all strains were constructed in this study.

### Plasmid construction

All plasmids used in this work are listed in Table 2. Primers were synthesized by Integrated DNA Technologies, Inc. (IDT, Coralville, IA) and are listed in Table 3. Genes were amplified from *S. enterica* or *P. aeruginosa* genomic DNA using KAPA Master Mix (Roche) following the manufacturer’s instructions. For cloning into pJM220 and pMQ30, protocols for Gibson assembly were followed. A high-efficiency cloning method described elsewhere (35, 36) was utilized to clone *acs* and *ackA* genes into pTEV18 or pTEV19 (IPTG-inducible overexpression vector). BspQI was purchased from New England Biolabs.

**TABLE 2:** Plasmids used in this study

Plasmid	Genotype	Source^*a*^
pTEV18	*lacI^+^ bla* +	(36)
pTEV19	*lacI^+^ bla* +	(36)
pJM220	*rhaSR*-P*_rhaBAD_ aacC^+^ bla* +	(33)
pMQ30	*sacB^+^ aacC^+^ura3*	(37)
Overexpression plasmids		
pPaAcsA	*P. aeruginosa acsA*^+^ cloned into pTEV18, *bla^+^*	
pSeAcs	*S. enterica acs*^+^ cloned into pTEV18, *bla^+^*	(36)
pPaAckA	*P. aeruginosa ackA*^+^ cloned into pTEV19, *bla^+^*	
pSeAckA	*S. enterica ackA*^+^ cloned into pTEV18, *bla^+^*	
Complementation plasmids		
pJM220-*acsA*	*P. aeruginosa acsA*^+^ cloned into pJM220, *bla^+^ aacC* +	
pJM220-*ackA-pta*	*P. aeruginosa ackA-pta*^+^ cloned into pJM220, *bla^+^ aacC* +	
Deletion construct plasmids		
pMQ30-*acsA*	1 kb upstream and downstream of *acsA* cloned into pMQ30	
pMQ30-*acsB*	1 kb upstream and downstream of *acsB* cloned into pMQ30	
pMQ30-*ackA-pta*	1 kb upstream and downstream of *ackA-pta* cloned into pMQ30	

^*a*^ Unless otherwise noted, all strains were constructed in this study.

**TABLE 3:** Primers used in this study

Primer name	Primer sequence 5′ → 3′
Overexpression primers (for insertion into pTEV18)	
5′ *acsA* pTEV18	NNGCTCTTCNTTCATGTCTGCGGCATCCCTGT
3′ *acsA* pTEV18	NNGCTCTTCNTTATCAGGCGGCCTGCATCGAG
5*′ acs* pTEV18	NNGCTCTTCNTTCATGAGCCAAACACATAAACAC
3′ *acs* pTEV18	NNGCTCTTCNTTATTATGACGGCATCGCGATGGC
5′ PA*ackA* pTEV18	NNGCTCTTCNTTCATGCCCTCACGCAACATACTGG
3′ PA*ackA* pTEV18	NNGCTCTTCNTTATCAGTCGAGCAGGGCCAG
5′ SE*ackA* pTEV18	NNGCTCTTCNTTCATGTCGAGTAAGTTAGTACTGGTTC
3′ SE*ackA* pTEV18	NNGCTCTTCNTTATCAGGCAGTCAGACGGC
Complementation primers (for insertion into pJM220)	
5′ *acsA*	CAGGAATTCCTCGAGAAGCTTAAGGAGGAACAGCTATGTCTGCGGCATCCCTGTA
3′ *acsA*	GCAAGGCCTTCGCGAGGTACTCAGGCGGCCTGCATCGAGC
5′ *ackA-pta*	CAGGAATTCCTCGAGAAGCTTAAGGAGGAACAGCTATGCCCTCACGCAACATACT
3′ *ackA-pta*	GCAAGGCCTTCGCGAGGTACTCAGGCCGGGGCCTGGGCGT
*P. aeruginosa* deletion primers (for cloning into pMQ30)	
*acsA* upstream F	ACGACGGCCAGTGCCAAGCTGTGATCCTGATGGTGGTGAT
*acsA* upstream R	CGTTTCGGATCAGGCGGAAGGGCTTTAACCTCGGTACGTG
*acsA* downstream F	CACGTACCGAGGTTAAAGCCCTTCCGCCTGATCCGAAACG
*acsA* downstream R	TACGAATTCGAGCTCGGTACTCATCGACGAGAGGATCGCA
*acsA* delcheck F	CGTGGCTGATGATCCTGCTGCT
*acsA* delcheck R	TTGACAGGCAGCGACATTTGTGC
*acsB* upstream F	ACGACGGCCAGTGCCAAGCTATGTCCATCGGCATCTCCAA
*acsB* upstream R	TTGAGGCGGTTGTCGATCAAGGCGGTTTCCTTTGTTTTTC
*acsB* downstream F	GAAAAACAAAGGAAACCGCCTTGATCGACAACCGCCTCAA
*acsB* downstream R	TACGAATTCGAGCTCGGTACCTTGCTGTCACGGCACGAAA
*acsB* delcheck F	CCAAGGCCTGATCGACTTCTTCC
*acsB* delcheck R	GCCGATCATCATGCTGCTGAAG
*ackA-pta* upstream F	ACGACGGCCAGTGCCAAGCTCATTCAGCGACACAACGTGC
*ackA-pta* upstream R	CACTGGGCGGCGTTCCTTCACAGTATGTTGCGTGAGGGCA
*ackA-pta* downstream F	TGCCCTCACGCAACATACTGTGAAGGAACGCCGCCCAGTG
*ackA-pta* downstream R	TATGACCATGATTACGAATTCGAGCAACTGGTCCTTGCGC
*ackA-pta* delcheck F	TGGGTCCTTGACGAAAACGAGC
*ackA-pta* delcheck R	ACGACAGCATCAAGGAGTCCTTGT
RT-qPCR primers	
5′ *acsA*	ACAACCTCGGCAACCTGATC
3′ *acsA*	TCCAGTAGTAGCCGTCCTCG
5′ *ackA*	TGATCTTCACCGGTGGCATC
3′ *ackA*	GATCACCAGTACCCGCGGAT
5′ *pta*	CAACCTGGTGTCGTCAGTGT
3′ *pta*	GTAGCTGATCATCGCGACCC
5′ *rpoS*	GTGGTCAAGGAGCTCAACGT
3′ *rpoS*	AAGTCACCCGTTCGTTCAGG

### RNA isolation

*P. aeruginosa* cultures (5 mL) were grown shaking at 37°C in MOPS minimal medium as described above. Once the OD_500_ reached 0.4, the culture was spun down (6,000 × *g*), flash-frozen, and stored at −80°C until processing. Anoxic cultures were spun down in a coy chamber. RNA was isolated using a protocol described elsewhere (38). In detail, frozen cell pellets were resuspended (150 μL) in boil solution (ethylenediaminetetraacetic acid [EDTA, 18 mM], SDS [0.025% vol/vol], 2-mercaptoethanol [1% vol/vol], formamide [95% vol/vol], and DEPC-treated water [up to final volume]) and heated (95°C) for 7 min. Cell suspensions were spun down (16,000 × *g*) for 5 min at room temperature, and supernatant (100 μL) was transferred to a new tube. Solution was diluted with DEPC-treated water (400 μL) and sodium acetate (50 μL, final concentration 0.3 M). Ice-cold ethanol (95% vol/vol, 1,650 μL) was added, and solutions were incubated at −80°C for 1 h. Solutions were centrifuged at 16,000 × *g* for 30 min at 4°C, and supernatant was decanted. RNA/DNA pellets were washed twice with 1,000 μL cold ethanol twice (70% vol/vol) and centrifuged at 8,000 × *g* for 5 min. The supernatant was poured off, tubes were briefly centrifuged, and the remaining liquid was aspirated. Pellets were air-dried upside down on Kimwipes for 30 min and resuspended in 100 μL RNase-free water. Tubes were centrifuged at 16,000 × *g* for 1 min (to rid of water insoluble material), and 90 μL was transferred to a new tube for subsequent DNase digestion. DNA was digested using TURBO DNase kit (Thermo Fisher Scientific) following the protocol for rigorous DNase treatment. After DNase treatment and DNase inactivation, mixtures were centrifuged at 10,000 × *g* for 1 min, and 100 μL of supernatant was transferred to a fresh tube. RNA was sodium acetate–ethanol precipitated as described above. RNA was resuspended in 100 μL, aliquoted into four tubes, and frozen at −80°C for later use. RNA quality and concentration were assessed using the Agilent 2100 Bioanalyzer with the RNA 6000 Nano kit (Georgia Genomics Facility, University of Georgia). Samples with RIN values over six were considered adequate for subsequent RT-qPCR experiments.

### cDNA synthesis and quantitative reverse transcription PCR

RNA samples were diluted in nuclease-free water (final concentration 10 ng/μL) to a final volume of 30 µL. Any remaining DNA from samples was removed using TURBO DNase kit (Thermo Fisher Scientific) following the manufacturer’s rigorous treatment protocol. After DNase digestion, RNA (18 µL) was used to synthesize cDNA using the iScript cDNA synthesis kit (Bio-Rad Laboratories) following the manufacturer’s protocol. A no-reverse transcriptase (RT) control was used for all samples. Synthesized cDNA (4.5 ng µL^−1^) was used as a template for PCR. For real-time PCR, 20 μL reaction mixtures were prepared in 96-well plates with 10 μL of FastSYBR Green Master Mix (Applied Biosystems), gene-specific primers (0.25 µM), and cDNA (15 ng or 3.33 μL of above diluted cDNA). The RT-qPCR was performed using an Azure Cielo real-time PCR system. The *P. aeruginosa rpoS* gene was used as an internal control, and gene-specific primers were designed using Primer 3 software. Each biological replicate (three for each strain) was analyzed in technical triplicate. No-RT controls were included for each biological replicate and each primer pair, as well as no-template controls. Cycle threshold (*C_T_*) data were normalized to *rpoS,* and these values were transformed using 2(e−Δ*C_T_*)/10^−6^ and reported as arbitrary gene expression units (EU) (39). Mean EU values of each technical triplicate were used to calculate the standard error of the mean (SEM) of three biological replicates using the Prism v10 software. Differences between genes in oxic and anoxic conditions were compared using Welch’s *t*-test in GraphPad Prism v10 software.

### Protein overproduction and purification

Plasmids coding for proteins of interest were transformed into *E. coli* C41 *pat::kan^+^* (λDE3). Overnight cultures (20 mL) of transformants were subcultured (2% [vol/vol]) into 1 L of LB medium containing ampicillin. Cultures were grown at 37°C with shaking to an optical density of 0.6 (OD_600nm_), after which plasmid expression was induced by the addition of IPTG (0.5 mM), and cultures were shaken at 37°C for 2 h. Cells were harvested by centrifugation at 6,000 × *g* for 10 min in a Beckman Coulter Avanti J-20 XOI refrigerated centrifuge with a JLA-8.1000 rotor at 4°C. Cell pellets were stored at −80°C until used. For purification, cell pellets were thawed and resuspended in 30 mL of buffer A (4-(2-hydroxyethyl)-1-piperazineethanesulfonic acid; HEPES [50 mM, pH 7.0 at 4°C], NaCl [500 mM], imidazole [20 mM], and glycerol [10% vol/vol]) containing lysozyme (1 mg/mL), DNase (1 μg/mL), and protease inhibitor phenylmethanesulfonyl fluoride (PMSF, 0.5 mM). Cells were lysed by four rounds of sonication on ice (1 min each, 2 s on, 2 s off, 60% duty) using a sonicator. Clarified lysates were obtained after centrifugation for 30 min at 4°C at 40,000 x *g* in a Beckman Coulter J2-HS centrifuge with a JA-20 rotor, followed by filtration of supernatant through a 0.45 μm filter (Millipore). Samples were applied at 4°C to a 1 mL HisPur nickel-nitrilotriacetic acid (Ni-NTA) resin (Thermo Scientific) that was pre-equilibrated with 10 column volumes (CV) of water and 10 CV of buffer A. After lysate was applied to the column, it was washed with 10 CV of buffer A and five CV of buffer B (HEPES [50 mM, pH 7.0 at 4°C], NaCl [500 mM], imidazole [60 mM], and glycerol [10% vol/vol]). Proteins were eluted with five CV of buffer C (HEPES [50 mM, pH 7.0 at 4°C], NaCl [500 mM], imidazole [500 mM], and glycerol [10% vol/vol]). Fractions were run on an SDS-PAGE gel, and fractions containing the desired protein were combined. Proteins were cleaved at 25°C for 3 h using a 1:50 mg:mg ratio of rTEV to target protein. After cleavage, proteins were dialyzed in buffer D (HEPES [50 mM, pH 7.5 at 4°C], NaCl [0.5 M], EDTA [1 mM], and glycerol [10% vol/vol]) and then twice in buffer A. After dialysis, cleaved proteins were applied to a pre-equilibrated 1 mL HisPur Ni-NTA resin. Proteins that did not bind to the column were collected and dialyzed against three buffers with decreasing concentrations of NaCl (400 mM, 250 mM, and 150 mM), with a final buffer composition of (HEPES [50 mM, pH 7.5 at 4°C], NaCl [0.15 M], and glycerol [20% vol/vol]). Protein concentrations were determined using a NanoDrop using the extinction coefficient and molecular weight of the protein (ExPASy ProtParam), and samples were flash frozen in liquid N_2_ and stored at −80°C until used.

### *In vitro* kinetics assays

Specific activity and kinetic parameters of Acs and AckA for acetate were quantified using an NADH consumption-coupled assay (40, 41). For Acs, reactions contained HEPES buffer (50 mM, pH 7.5 at 25°C), TCEP (1 mM), ATP (2.5 mM), coenzyme A (CoASH, 0.5 mM), MgCl2 (5 mM), phosphoenol pyruvate (3 mM), NADH (0.1 mM), pyruvate kinase (1 U), myokinase (5 U), lactate dehydrogenase (1.5 U), acetate (concentration in figures), and Acs (0.03 µM). For AckA, reactions contained HEPES buffer (50 mM, pH 7.5 at 25°C), TCEP (1 mM), ATP (20 mM), MgCl2 (25 mM), phosphoenol pyruvate (5 mM), NADH (0.1 mM), pyruvate kinase (2 U), lactate dehydrogenase (3 U), acetate (concentration in figures), and AckA (0.03 µM). All reactions were started by the addition of acetate. Changes in NADH absorbance were monitored at 340 nm for 10 min at 30 s intervals in a 96-well plate format using a SpectraMax Plus UV-visible spectrophotometer (Molecular Devices). Enzyme activities were calculated as described (42). Activity was calculated from the slope of the linear range of (ΔA_340_ s^−1^) using Beer’s law (*A* = [ε]*lc*) with a path length of 1 cm and the molar extinction coefficient of NADH (6,220 M^−1^ cm^−1^). To calculate kinetic parameters, graphs of initial velocity (μM s^−1^) versus substrate concentration (μM) were plotted using Prism v10 (GraphPad). Data were fitted to the Michaelis–Menten kinetics model to determine *K_m_* and *V*_max_. The turnover number (*k*_cat_) was determined using the following equation: *V*_max_ *= k*_cat_[*E*], where [*E*] was the concentration of protein added. All spectrophotometric assays mentioned above were completed thrice, each in technical quadruplicate with a representative data set shown. Error bars represent standard deviation. Tables in Fig. 5 represent the mean and standard deviation of three independent experiments.
